# Analysis of *Bos taurus* and *Sus scrofa* X and Y chromosome transcriptome highlights reproductive driver genes

**DOI:** 10.18632/oncotarget.17081

**Published:** 2017-04-13

**Authors:** Faheem Ahmed Khan, Hui Liu, Hao Zhou, Kai Wang, Muhammad Tahir Ul Qamar, Nuruliarizki Shinta Pandupuspitasari, Zhang Shujun

**Affiliations:** ^1^ Key Laboratory of Agricultural Animal Genetics, Breeding and Reproduction, Ministry of Education China, College of Animal Science and Technology Huazhong Agricultural University, Wuhan, China; ^2^ College of Informatics, Huazhong Agricultural University, Wuhan, China; ^3^ The Center for Biomedical Research, Tongji Hospital, Tongji Medical College, Huazhong University of Science and Technology, Wuhan, China

**Keywords:** spermatogenesis, spermiogenesis, fertilization, WGCNA, reproduction

## Abstract

The biology of sperm, its capability of fertilizing an egg and its role in sex ratio are the major biological questions in reproductive biology. To answer these question we integrated X and Y chromosome transcriptome across different species: *Bos taurus* and *Sus scrofa* and identified reproductive driver genes based on Weighted Gene Co-Expression Network Analysis (WGCNA) algorithm. Our strategy resulted in 11007 and 10445 unique genes consisting of 9 and 11 reproductive modules in *Bos taurus* and *Sus scrofa*, respectively. The consensus module calculation yields an overall 167 overlapped genes which were mapped to 846 DEGs in *Bos taurus* to finally get a list of 67 dual feature genes. We develop gene co-expression network of selected 67 genes that consists of 58 nodes (27 down-regulated and 31 up-regulated genes) enriched to 66 GO biological process (BP) including 6 GO annotations related to reproduction and two KEGG pathways. Moreover, we searched significantly related TF (ISRE, AP1FJ, RP58, CREL) and miRNAs (bta-miR-181a, bta-miR-17-5p, bta-miR-146b, bta-miR-146a) which targeted the genes in co-expression network. In addition we performed genetic analysis including phylogenetic, functional domain identification, epigenetic modifications, mutation analysis of the most important reproductive driver genes PRM1, PPP2R2B and PAFAH1B1 and finally performed a protein docking analysis to visualize their therapeutic and gene expression regulation ability.

## INTRODUCTION

The sex chromosomes XY or ZW in case of mammals or birds varies both phylogenetically and structurally among different taxonomical classes with many similar features and is under selective pressure that shapes their evolution [1, 2, 3]. The karyotypes shows that the hemizygous chromosome Y or W as short, heterochromatic containing few genes while on the other hand the homozygous chromosomes X or Z is of same size to that of autosomes and contains similar amount of genes although it tends to cluster functionally similar genes together most of which are enriched for reproduction and differentiation [4].

**Figure 1:**
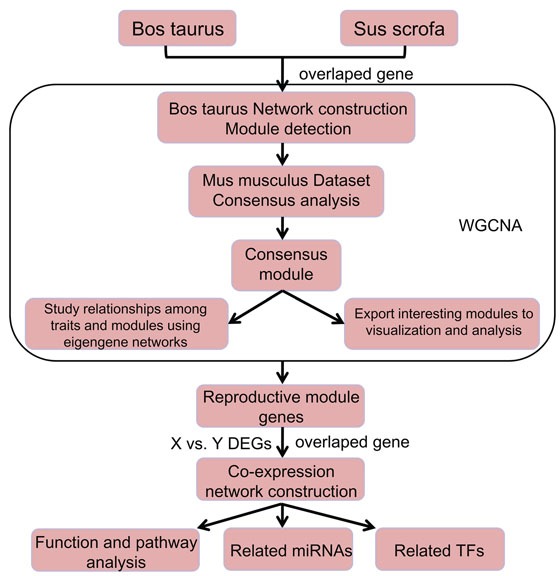
A multi-step Strategy The schematic diagram for a multi-step strategy to identify reproductive driver genes.

Reproduction is the fundamental biological process by which animals produce their offspring. In mammals it is done by sexual reproduction where a haploid sperm from male fertilizes a haploid oocyte to restore the chromosome number. To explore the reproductive driver genes that can improve the reproductive health and fertility is at the core of reproductive biology. The recent advances in sequencing technologies and its analysis have a strong potential to find out genes that can improve the overall reproductive performance of farm animals including cow and pig. To this end, we first integrated X and Y chromosome transcriptome across different species: *Bos taurus* and *Sus scrofa* to identify reproductive driver genes based on Weighted Gene Co-Expression Network Analysis (WGCNA) algorithm. The schematic diagram for a multi-step strategy to identify reproductive driver genes is shown in Figure 1.

The paternal genetic material across different species is transferred to oocyte by sperm after several dramatic morphological and physical changes [5]. A global transcriptional silencing occurs after meiosis as sperm nuclear basic proteins replaces somatic histones and cause tight DNA compaction [6, 7], and also most of the cytoplasmic material including ribosomes are discarded [8]. At this stage post translational mediators during the process of morphogenesis and motility of sperm become most important as there is no global transcription and translation [5]. The sperm motility is crucial for its ability to fertilize oocyte where PP1 a sperm specific phosphatase in mouse [9, 10] and GSP 3/4 in *C. elegans* have been shown important phosphatases for sperm motility [5]. The process of male and female gametes i.e. sperm and oocyte to unite and form a zygote is termed as fertilization. The sperm passes several developmental stages before it can reach oocyte in the female body to fertilize it. One such process is when the spermatids start to develop tails which will help them to swim in female reproductive tract to reach oocyte. This process is under strict control of gene expression. It has been observed that a change in gene expression level preferentially benefits X or Y chromosome bearing spermatozoa to develop tails and causes a skewed female or male sex ratio. In this scenario, genes that are responsible for sperm maturity takes a central position as candidate genes for sex ratio control. The WGCNA analysis also determines a cohort of genes that determine the process of sperm maturity and their preferential selection for tail development of specific X or Y chromosome bearing spermatozoa hence providing grounds for their selection as candidate genes in sex ratio control [5].

The genes involved in sexual development, spermatogenesis, spermatid development and differentiation and pregnancy are of vital importance in reproductive biology. Spermatogenesis is a complex process that is involved in the development of highly differentiated haploid sperms from diploid spermatogonial stem cells that is further divided into three phases of almost equal length, the spermatogoniogenesis [11], meiosis that generates four haploid spermatids [12] and spermiogenesis a process of dramatic morphological changes from spermatid to mature spermatozoa [13]. Spermatogenesis sequence of happenings is well defined in mammals and takes place in seminiferous tubules of the testes that is initiated at puberty, a continuous process in reproductively active adults to maintain the continuous sperm production with a fully controlled timing, in mouse it is 35 days [14].

The present study explores the integrated X and Y chromosome trancriptome across *Bos taurus* and *Sus scrofa* and identified important reproductive driver genes including PRM1, PPP2R2B and PAFAH1B1. Interestingly various immune related genes are also expressed together with genes involved in reproduction suggesting its probable role in reproduction specifically in preferential selection of X and Y chromosome bearing spermatozoa before fertilization. The preferential selection of X or Y chromosome bearing spermatozoa by female can have a profound importance for farm animals where farmers can select animals gender without the risk of losing subsequent fertilization ability of sperm in female oviduct that are exposed to sorted sperm [15]. Furthermore, the present study conducts complete genetic analysis of the genes to figure out its comprehensive role in reproduction. Moreover, protein docking analysis is performed to assess its therapeutic potential in case of infertility or its potential use as sex ratio control as well as to regulate the gene expression profile. Hence the present study is designed to explore an important economic aspect in animal reproduction with wide applicability.

## RESULTS

### Data pre-processing

In present study, we got 11007 and 10445 unique genes in *Bos taurus* and *Sus scrofa*, respectively, where we normalized expression data with Limma package. Expression data, before and after normalization can be found in Supplementary Table 1 (GSE47139-before.txt”, “GSE47139-norm.txt”, “xy-before.txt” and “xy-norm.txt) and Supplementary Figure 1. *Bos taurus* and *Sus scrofa* expression data shared 555 homologous genes which were listed in Supplementary Table 2.

### Consensus modules identification of *Bos taurus* and *Sus scrofa*

#### Definition of adjacency function

**Figure 2:**
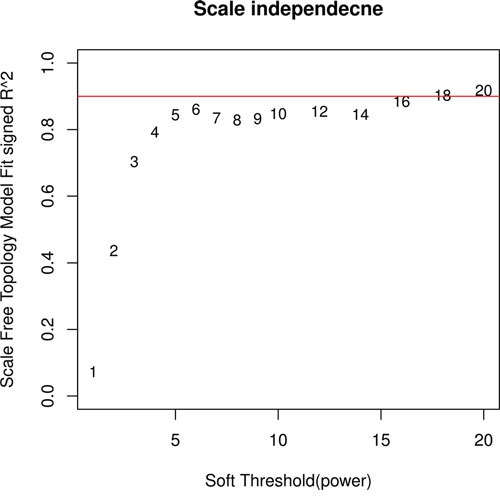
Adjacency matrix weight parameter power plot The horizontal axis represents the weight parameter power, the vertical axis represents the square of the correlation coefficient between log (k) and log (p (k)).

In order to satisfy the preconditions of scale-free network distribution, we need to explore the value of the adjacency matrix weight parameter: power. The power plot was shown in Figure 2. The higher the square of the correlation coefficient is, the closer the network is to the network-free distribution. We choose power = 20 as the power cutoff, when the square of the correlation coefficient reached 0.9 for the first time.

#### Identification consensus modules of *Bos taurus*

**Figure 3:**
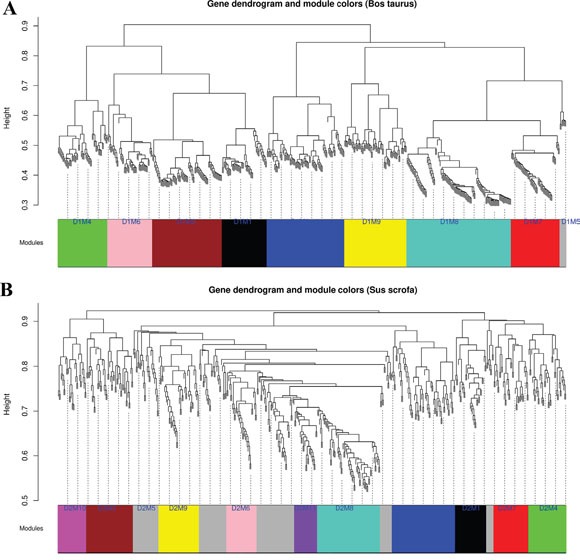
Module clustering tree of *Bos taurus* **(A)** and *Sus scrofa* **(B)**. Different colours in module bar mean different modules. D1 and D2 refer to *Bos taurus* and *Sus scrofa* datasets, respectively. M1 to M11 mean different module numbers.

We regarded *Bos taurus* gene expression data as training dataset, constructed network and mined gene modules based on 555 shared genes in *Bos taurus*. Firstly, the coefficients of dissimilarity between genes were calculated, and the system clustering tree was obtained. Then cut the clustering tree according to standard for hybrid dynamic shear trees. We set minimum number of genes per module to 30 genes and the pruning height to cutHeight = 0.9. We finally got 9 different modules in *Bos taurus* gene expression data, shown in Figure 3A.

#### Screening consensus modules in *Sus scrofa*

**Figure 4:**
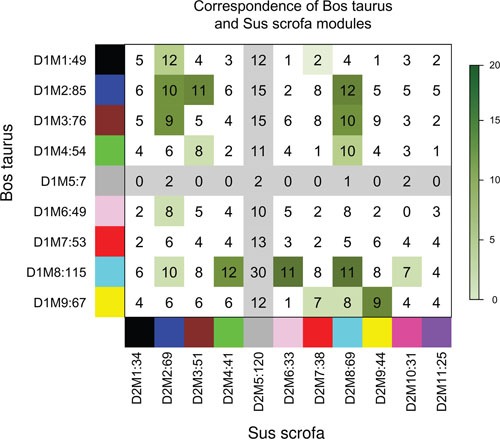
Correspondence of *Bos taurus*-specific modules and *Sus scrofa*-specific modules Numbers in boxes refer to overlapped genes in every two modules. Color bar in the left means significance p value of module consensus,0-20 represents –log_2_(p value).

We also screened gene modules in *Sus scrofa* by using same method and parameters as in *Bos taurus*. We obtained 11 gene modules in *Sus scrofa*, 2 more modules than that in *Bos taurus*, as shown in Figure 3B. Then we calculate consensus of every two modules in *Bos taurus* and *Sus scrofa*. The consistency of modules was displayed in Figure 4. Information for each module and overlapped genes were listed in Table 1. Genes and their corresponding colors of *Bos taurus* and *Sus scrofa* can be found in Supplementary Table 3.

**Table 1:** Characterization of *Bos taurus* and *Sus scrofa* modules

Bos taurus Module	Sus scrofa Module	Module color	Bos taurus module gene count	Sus scrofa module gene count	Overlap number	Overlap p value
D1M1	D2M2	black-blue	49	69	12	0.00817
D1M1	D2M7	black-red	49	38	2	0.0423
D1M2	D2M2	blue-blue	85	69	10	0.00846
D1M2	D2M3	blue-brown	85	50	11	0.00679
D1M2	D2M8	blue-turquoise	85	69	12	0.000142
D1M3	D2M2	brown-blue	76	69	9	0.00035
D1M3	D2M8	brown-turquoise	76	69	10	0.000482
D1M4	D2M3	green-brown	54	50	8	0.0116
D1M4	D2M8	green-turquoise	54	69	10	0.0047
D1M6	D2M2	pink-blue	49	69	8	0.0144
D1M8	D2M2	turquoise-blue	114	69	10	0.0102
D1M8	D2M4	turquoise-green	114	41	12	0.00047
D1M8	D2M6	turquoise-pink	114	33	11	0.008203
D1M8	D2M8	turquoise-turquoise	114	69	11	0.00848
D1M8	D2M10	turquoise-magenta	114	31	7	0.00134
D1M9	D2M7	yellow-red	68	38	7	0.00123
D1M9	D2M8	yellow-turquoise	68	69	8	0.00136
D1M9	D2M9	yellow-yellow	68	44	9	0.000435

We collected significantly overlapped genes in consensus modules for further analysis, that is to say, a total of 167 genes in column 6 of Table 1, which were listed in Supplementary Table 4.

### Gene significance analysis

**Figure 5:**
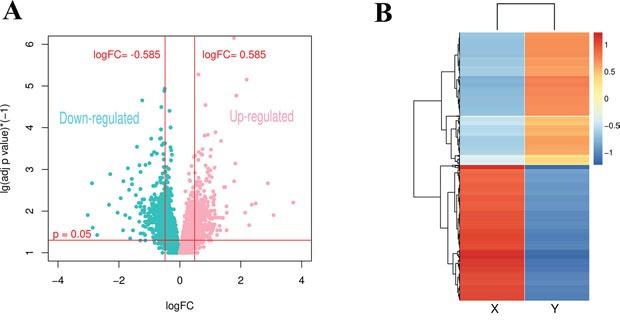
**(A)** Volcano plot of statistic test for gene expression in *Bos taurus*. **(B)** Heatmap of DEGs in *Bos taurus*.

A gene set with 846 gene which met cutoff (p<0.05 and |log2FC|>0.585) were screened as DEGs in *Bos taurus* by using Limma package in R language, statistic test volcano plot and heatmap were shown as Figure 5A and 5B, DEGs were listed in Supplementary Table 5. Then we mapped DEGs into 167 module genes selected previously, we finally found 67 genes which had dual features: significantly expressed between X and Y chromosome transcriptome, as well as significantly overlapped in consensus modules. The 67 dual featured genes were included into further analysis (gene list can be found in Supplementary Table 6).

### Construction of co-expression network of shared genes

**Figure 6:**
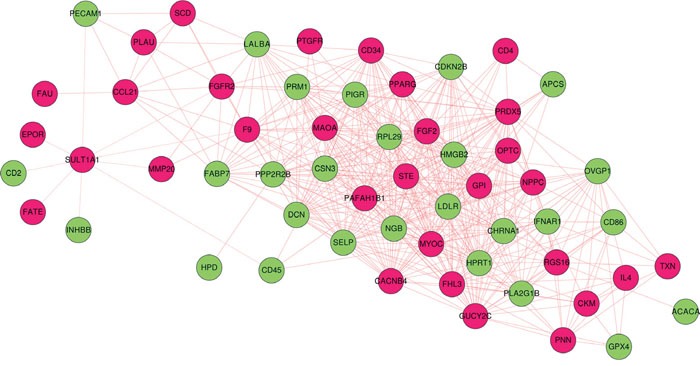
Co-expression network of 67 selected genes Red and green nodes refer to up and down regulated genes in *Bos taurus*; dash and solid edge mean negative and positive correlation coefficient.

We calculated expression correlation coefficient of 67 interested genes and ticked out gene pairs whose correlation coefficient below 0.8 (correlation coefficient matrix was in Supplementary Table 7), finally constructed co-expression network among 67 genes, as shown in Figure 6. The co-expression network was consist of 58 nodes (27 down-regulated and 31 up-regulated genes) and 497 edges.

### Functional and KEGG pathway analysis of the network genes

**Figure 7:**
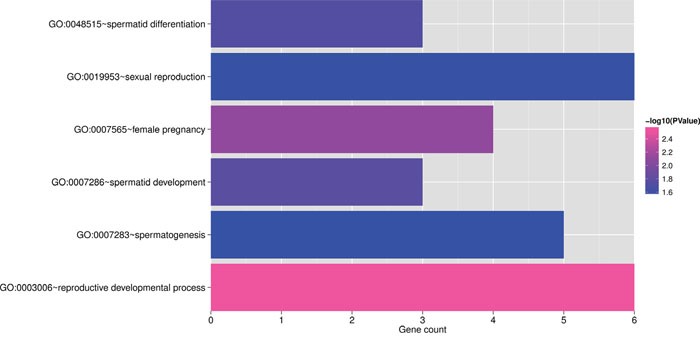
Barplot for reproduction related GO annotations The horizontal axis represents gene count, the vertical axis represents GO annotations, color bar shows –log (p), where p is enrichment significant.

We enriched genes in the co-expression network to GO and KEGG pathway, and got 66 significantly related GO biology process (BP) annotation and 2 KEGG pathways with cutoff of p<0.05. We extracted 6 GO annotations which were related to reproduction to display, as shown in Figure 7, listed in Table 2-1. The KEGG pathway analysis list can be found in Table 2-2 The complete table for GO annotations can be found in Supplementary Table 8.

**Table 2-1:** Reproduction related GO annotations list

Term	Count	P Value	Genes
GO:0003006˜reproductive developmental process	6	0.002729	INHBB, HMGB2, PAFAH1B1, EPOR, PRM1, PPP2R2B
GO:0019953˜sexual reproduction	6	0.026424	OVGP1, HMGB2, GPX4, PAFAH1B1, PRM1, PPP2R2B
GO:0007283˜spermatogenesis	5	0.026725	HMGB2, GPX4, PAFAH1B1, PRM1, PPP2R2B
GO:0007565˜female pregnancy	4	0.00777	OVGP1, EPOR, PLAU, RPL29
GO:0007286˜spermatid development	3	0.016952	PAFAH1B1, PRM1, PPP2R2B
GO:0048515˜spermatid differentiation	3	0.018774	PAFAH1B1, PRM1, PPP2R2B

**Table 2-2:** KEGG pathway annotations list

Term	Count	PValue	Genes
hsa04514:Cell adhesion molecules (CAMs)	6	0.002756	SELP, CD86, CD34, PECAM1, CD2, CD4
hsa04640:Hematopoietic cell lineage	5	0.003632	IL4, CD34, CD2, EPOR, CD4

### Related miRNA and TF search

**Figure 8:**
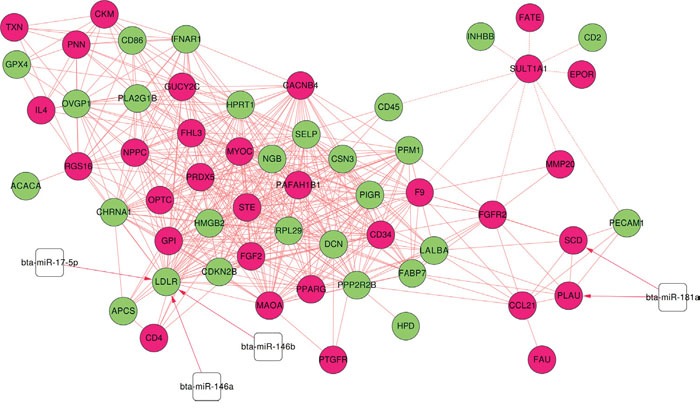
miRNA-targeted co-expression network Red and green nodes refer to up and down regulated genes in *Bos taurus*, white square is miRNAs; dash and solid edge mean negative and positive correlation coefficient, red line with arrow mean miRNAs target genes.

We searched significantly related TF using The Database for Annotation, Visualization and Integrated Discovery (DAVID) v6.8 and got 4 related TFs: ISRE, AP1FJ, RP58, CREL, see Table 3. In addition, we extracted 4 related miRNAs which targeted genes in co-expression network from miRTarBase (2016 version): bta-miR-181a, bta-miR-17-5p, bta-miR-146b, bta-miR-146a, listed in Supplementary Table 9. We integrated miRNA and their target information into co-expression network to construct miRNA-targeted co-expression network, as shown in Figure 8.

**Table 3:** Related TF list

Category	Term	Count	P Value	Genes
UCSC_TFBS	ISRE	27	0.005515	FGFR2, LALBA, PPARG, PRDX5, CACNB4, PNN, CDKN2B, CCL21, SULT1A1, CD2, PLA2G1B, FAU, PAFAH1B1, CD4, CHRNA1, PPP2R2B, IL4, SELP, MAOA, ACACA, F9, PIGR, RGS16, IFNAR1, GPI, CD86, EPOR
UCSC_TFBS	AP1FJ	19	0.008982	FGFR2, FHL3, ACACA, CACNB4, PTGFR, RPL29, MMP20, CD86, CKM, CDKN2B, CD34, CCL21, GPX4, PECAM1, EPOR, PAFAH1B1, CD4, NGB, PPP2R2B
UCSC_TFBS	RP58	30	0.032744	FGFR2, LALBA, PPARG, FHL3, DCN, CACNB4, MMP20, CDKN2B, GPX4, PLA2G1B, FAU, PAFAH1B1, CHRNA1, PPP2R2B, FGF2, OPTC, SELP, MAOA, ACACA, F9, PIGR, GUCY2C, RGS16, PTGFR, INHBB, GPI, CD34, PECAM1, NPPC, EPOR
UCSC_TFBS	CREL	12	0.044246	IL4, MMP20, CD34, GPX4, PPARG, FHL3, ACACA, EPOR, CD4, CACNB4, PIGR, FGF2

It can be observed that RP58, ISRE and APIFJ are significantly related to 30, 27 and 19 genes respectively including PAFAH1B1 and PPP2R2B. PLAU, a gene responsible for creating suitable environment for embryo implantation and SCD, a gene responsible for fatty acids have a miRNA binding sites for bta-miR-181a. LDLR that has a demonstrated role in sperm storage tubules in hen [16] and its lack of presence in female mouse is associated with low fertility [17]. We have found 3 miRNA binding sites including bta-miR-17-5p, bta-miR-146b, bta-miR-146a associated with LDLR that can prove beneficial target for improving fertility. The expression of LDLR, PLAU and SCD in sperm transcriptome suggests it's crucial role in fertilization and activating energy related pathways.

### Association of reproductive genes with immunity and synaptic transmission genes

The reproduction have several predominant biological processes like spermatogenesis, sexual development and pregnancy. The genes associated with these processes are co-expressed with genes associated with immunity in respective modules indicating interesting cross talk among reproductive and immune related genes. The reproductive driver genes PPP2R2B and PRM1 lies in brown module along with important immunity related genes including TREM-1, TNF and TRAF-6, whereas PAFAH1B1 lies in blue module along with immune related genes IL4, IL6 etc. in *Bos taurus* suggesting a cross talk between reproductive and immune related genes.

It is worth noting that the reproductive driver gene PAFAH1B1 is also associated with synaptic transmission along with other genes including MAOA, CACNB4, CHRNA1, FGF2 suggesting its pivotal role as neuro-transmitter that activates the receptors of another neuron indicating functional role in reproductive endocrinology.

### Genetic analysis of vital reproduction genes across *Bos taurus* and *Sus scrofa*

We found 6 reproduction related GO annotations (listed in Table 2-1) which involved 10 genes: PPP2R2B, PAFAH1B1, PRM1, HMGB2, OVGP1, GPX4, EPOR, PLAU, RPL29, INHBB. Among the 10 genes, PPP2R2B, PRM1 and PAFAH1B1 participated in 5 reproduction related GO annotations at the same time. Therefore, PPP2R2B, PRM1 and PAFAH1B1 are considered as vital reproduction related genes. We did a series of genetic analysis for the selected three genes: PPP2R2B, PRM1 and PAFAH1B1.

#### Genetic phylogenetic tree of PPP2R2B, PRM1 and PAFAH1B1 in *Bos taurus* and *Sus scrofa*

**Figure 9:**
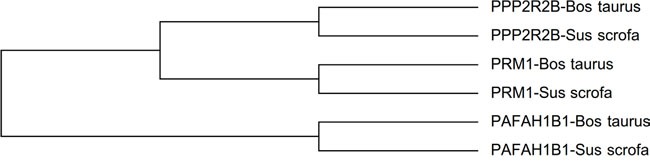
Phylogenetic tree of PPP2R2B, PRM1 and PAFAH1B1 in *Bos taurus* and *Sus scrofa*

We downloaded protein sequences of these three genes in *Bos taurus* and *Sus scrofa* from NCBI database (Supplementary Table 10), and aligned them with ClustalW in Mega6 (18), and made a phylogenetic tree as shown in Figure 9. It can seen from the phylogenetic tree that genes with the same names in *Bos taurus* and *Sus scrofa* have a closer relationship in evolution.

#### Consensus sequences of PPP2R2B, PRM1 and PAFAH1B1 in *Bos taurus* and *Sus scrofa*

**Figure 10:**
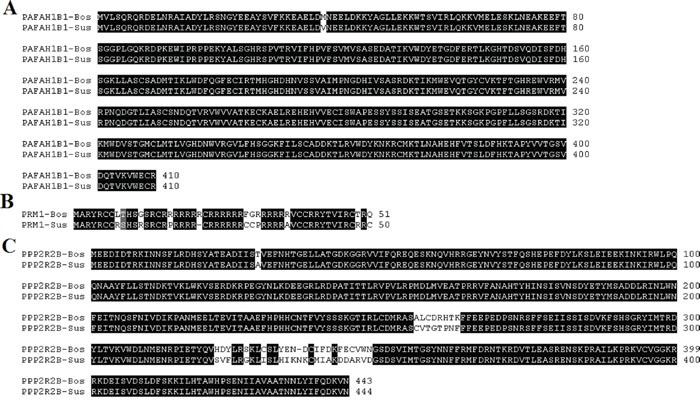
Consensus sequences of PAFAH1B1 **(A)** PRM1 **(B)** and PPP2R2B **(C)** in *Bos taurus* and *Sus scrofa*. Sequences with black background are consensus sequences.

The calculated order of the most frequent residues that is of nucleotides or amino acids found at each position in sequence alignment is termed as consensus sequence. The related sequences in the multiple alignment are compared to each other and similar sequence motifs are calculated. We aligned downloaded protein sequences with ClustalW in Mega6 and found consensus sequences of PPP2R2B, PRM1 and PAFAH1B1 across *Bos taurus* and *Sus scrofa*, results were shown in Figure 10. The genes with the same names in *Bos taurus* and *Sus scrofa* have a higher consistency in the sequence.

#### Domains in PPP2R2B, PRM1 and PAFAH1B1

**Figure 11:**
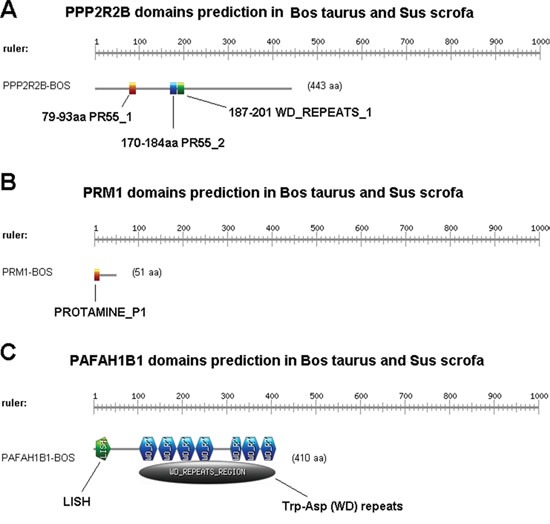
The visualization result of Domain in PPP2R2B, PRM1 and PAFAH1B1

In our present integrated transcriptome study of *Bos taurus* and *Sus scrofa* we identified PPP2R2B, PAFAH1B1 and PRM1 as candidate genes involved in spermatid differentiation and development. We then searched domains in PPP2R2B, PRM1 and PAFAH1B1 with InterProScan [19] in EBI, and finally got 3, 1 and 2 domains, respectively. Information of each domain was listed in Table 4. The visualization result are shown in Figure 11.

**Table 4:** Domain information list of PPP2R2B, PRM1 and PAFAH1B1

**PPP2R2B**		
**Postion**	**Domain name**	**Domain full name**
79-93	PR55_1	Protein phosphatase 2A regulatory subunit PR55 signature 1
170-184	PR55_2	Protein phosphatase 2A regulatory subunit PR55 signature 2
187-201	WD_REPEATS_1	Trp-Asp (WD) repeats signature
**PRM1**		
**Postion**	**Domain name**	**Domain full name**
2-13	PROTAMINE_P1	Protamine P1 signature
**PAFAH1B1**		
**Postion**	**Domain name**	**Domain full name**
7-39	LISH	LIS1 homology (LisH) motif
104-410	WD_REPEATS_2	Trp-Asp (WD) repeats

“PR55 a domain in PPP2R2B” has diverse functions including substrate recognition, target an enzyme to correct subcellular localization, have a role in spindle cell assembly and centrosome attachment to nuclei. The expression of PPP2R2B hence indicates its critical role in reproduction especially spermatogenesis. Apart from PR55, WD-REPEATS domain is also present in PPP2R2B. PRM1 contains PROTAMINE domain which plays important roles sperm chromatin is substituted for histones during haploid spermatogenic phase. The sperm DNA is packed into a highly condensed, stable and inactive complex by protamines hence maintaining sperm resistant to lethal mutations. LISH and WD-REPEATS domains correspond to PAFAH1B1. In present study we found PAFAH1B1 as an important phosphatase for sperm motility and its fertilizing capability hence regarded as critical factor in male fertility. The 33-residue LIS1 homology (LisH) motif is found in eukaryotic intracellular proteins that is involved in microtubule dynamics, cell migration, nucleokinesis and most importantly in chromosome segregation when we look for its function in reproduction.

#### Epigenetic predictions for PPP2R2B, PRM1 and PAFAH1B1 in *Bos taurus* and *Sus scrofa*

**Figure 12:**
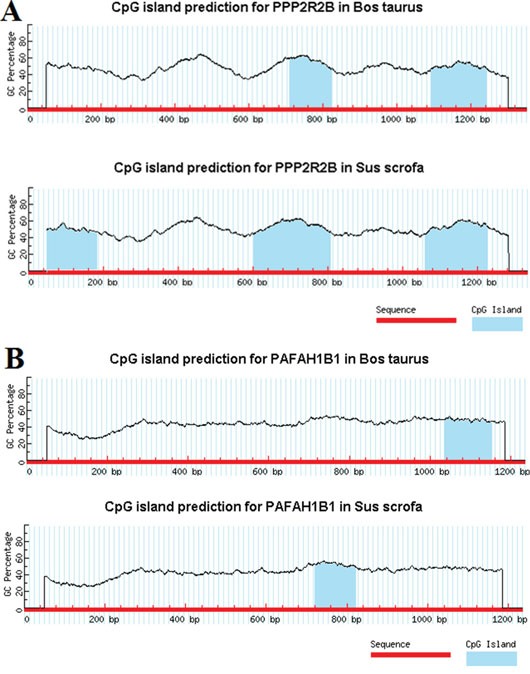
CpG islands in PPP2R2B and PAFAH1B1 in *Bos taurus* and *Sus scrofa*

Cytosines in CpG dinucleotides can be methylated to form 5-methylcytosine. In mammals, a methylating cytosine within a gene can change its expression pattern, hence regulating gene epigenetically. We searched CpG islands of PPP2R2B, PRM1 and PAFAH1B1 in *Bos taurus* and *Sus scrofa*. In our prediction, we used Methprimer [20] to identify CpG islands. CpG islands are defined as sequence ranges where the Obs/Exp value is greater than 0.6 and the GC content is greater than 60%. We found no CpG islands in PRM1. The results of PPP2R2B and PAFAH1B1 are shown in Figure 12.

#### Mutations in PPP2R2B, PRM1 and PAFAH1B1

We searched SNPs in PPP2R2B, PRM1 and PAFAH1B1 from dbSNP, shown in Table 5. In addition, we also searched CNVs of each gene from Database of Genomic Variants (DGV), listed in Table 6. A total of five SNPs were found in PPP2R2B including one missense rs11547494, while PAFAH1B1 was found to have three SNPs rs12143448254, rs12143448454, rs12143448654, all of which are missense from dbSNP database. There was no SNP found in PRM1 in dbSNP database.

**Table 5:** Sequence variations from dbSNP

**PPP2R2B**			
**SNP ID**	**Chr 05 pos**	**Sequence Context**	**Type**
rs11547494	146,701,106(-)	CACGG(G/T)AGAAT	nc-transcript-variant, reference, missense
rs160967	146,803,933(+)	gaggc(C/T)gaggc	intron-variant
rs160968	146,829,614(+)	TGGCC(C/T)ATTTC	intron-variant
rs160969	146,837,254(-)	atcat(C/G/T)tcatg	intron-variant
rs160970	146,836,216(-)	AAAAC(A/G)TAACC	intron-variant
**PAFAH1B1**			
**SNP ID**	**Chr 17 pos**	**Sequence Context**	**Type**
rs121434482 5 4	2,670,209(+)	AGGAC(A/G)TACAG	reference, missense
rs121434484 5 4	2,670,268(+)	CCTGT(C/T)CTGCA	reference, missense
rs121434486 5 4	2,665,431(+)	AGTTT(C/T)TAAAA	reference, missense

**Table 6:** Structural variations from Database of Genomic Variants (DGV)

**PPP2R2B**			
**Variant ID**	**Type**	**Subtype**	**PubMed ID**
dgv1656e212	CNV	Loss	25503493
dgv356n21	CNV	Loss	19592680
esv1159529	CNV	Insertion	17803354
esv2053966	CNV	Deletion	18987734
esv2422173	CNV	Deletion	20811451
esv2659662	CNV	Deletion	23128226
esv2660911	CNV	Deletion	23128226
esv2664858	CNV	Deletion	23128226
esv26695	CNV	Loss	19812545
esv2670593	CNV	Deletion	23128226
esv2672689	CNV	Deletion	23128226
esv2675913	CNV	Deletion	23128226
esv2730881	CNV	Deletion	23290073
esv2730882	CNV	Deletion	23290073
esv2730883	CNV	Deletion	23290073
esv2759385	CNV	Loss	17122850
esv2763505	CNV	Loss	21179565
esv3304864	CNV	mobile element insertion	20981092
esv3306710	CNV	mobile element insertion	20981092
esv3310476	CNV	novel sequence insertion	20981092
esv3324641	CNV	Insertion	20981092
esv3394455	CNV	Insertion	20981092
esv3427676	CNV	Duplication	20981092
esv3429928	CNV	Insertion	20981092
esv3444421	CNV	Insertion	20981092
esv3570481	CNV	Loss	25503493
esv3570482	CNV	Loss	25503493
esv3570485	CNV	Loss	25503493
esv3607080	CNV	Loss	21293372
esv3607081	CNV	Loss	21293372
esv3607083	CNV	Loss	21293372
esv3607084	CNV	Loss	21293372
esv3607085	CNV	Loss	21293372
esv3607086	CNV	Loss	21293372
esv988317	CNV	Insertion	20482838
nsv1117590	CNV	Deletion	24896259
nsv1145843	CNV	Deletion	26484159
nsv327242	CNV	Insertion	16902084
nsv328338	CNV	Deletion	16902084
nsv499741	CNV	Loss	21111241
nsv5052	CNV	Insertion	18451855
nsv5053	CNV	Deletion	18451855
nsv514327	CNV	Loss	21397061
nsv519847	CNV	gain+loss	19592680
nsv528827	CNV	Loss	19592680
nsv599938	CNV	Gain	21841781
nsv823286	CNV	Loss	20364138
nsv969003	CNV	Duplication	23825009
**PRM1**			
**Variant ID**	**Type**	**Subtype**	**PubMed ID**
nsv1040428	CNV	Gain	25217958
nsv571453	CNV	Loss	21841781
**PAFAH1B1**			
**Variant ID**	**Type**	**Subtype**	**PubMed ID**
esv2660206	CNV	Deletion	23128226
esv2664869	CNV	Deletion	23128226
esv2715502	CNV	Deletion	23290073
esv2715503	CNV	Deletion	23290073
esv275250	CNV	gain+loss	21479260
esv3572343	CNV	Gain	25503493
esv3582486	CNV	Loss	25503493
esv3639714	CNV	Loss	21293372
esv3639715	CNV	Loss	21293372
nsv1055844	CNV	Gain	25217958
nsv1065489	CNV	Gain	25217958
nsv1070794	CNV	Deletion	25765185
nsv1123087	CNV	Deletion	24896259
nsv516756	CNV	Gain	19592680
nsv833339	CNV	Loss	17160897
nsv954919	CNV	Deletion	24416366

### Protein docking analysis of vital reproduction genes

**Figure 13:**
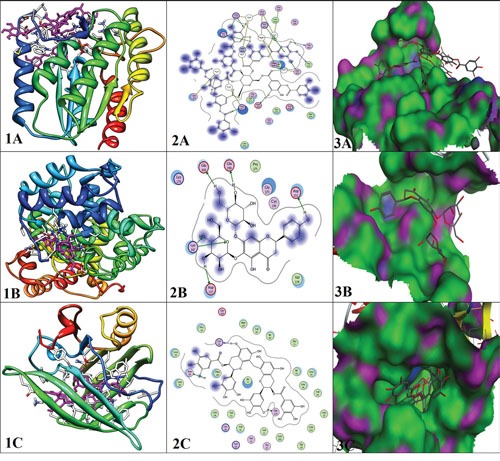
Interaction analys is of docked proteins; **(A)** (1, 2, 3) PAFAH1B1, **(B)** (1, 2, 3) PPP2R2B, **(C)** (1, 2, 3) PRM1.

Protein structures of PAFAH1B1 (PDB ID: 3DT6), PPP2R2B (PDB ID: 4MEW) and PRM1 (PDB ID: 4Y0Q) were downloaded from PDB and docked against MPD3 database ready to dock library using MOE docking tool. Ten conformations were provided by MOE for each ligand. These conformations were arranged according to S score. From 2500 MPD3 database ligands, only top seven individual conformations for every ligand which showed minimum S score and RMSD value were chosen. Along with minimum S score, these selected ligands also showed strong binding with targeted proteins. Among the strongly bonded selected ligands Tannic Acid, 5,6,7,4'-Tetrahydroxyflavanone 6,7-diglucoside, Oolonghomobisflavan-A showed most favorable results with PAFAH1B, PPP2R2B, and PRM1 respectively. The detailed results of docking including information about interacting residues are shown in Table 7 along with interacting residue figures in Figure 13 where 1A, 1B, 1C are showing protein-ligand complexes (ligand is in magenta color), 2A, 2B, 2C showing 2D docked interactions of ligand-protein and 3A, 3B, 3C are showing binding pocket mode of proteins.

**Table 7:** Docking results of vital reproductive genes

MPD3 ID	Ligands	S-Score	RMSD Value	Interacting Residues
**PAFAH1B1**				
2068	Tannic Acid	-21.31	3.61	Ser76, Leu194, Asn104, His79, Arg141, His106, Gln78, Thr12, Pro13, Gln15, Gln50, Gly73, Val17, Asp20, Arg22, Asp16, Trp23, Met24, Gly21, Leu26
**PPP2R2B**				
778	5,6,7,4'- Tetrahydroxyflavanone 6,7-diglucoside	-18.41	2.10	Asp213, Cys174, Gly173, Pro175, Glu299, Glu300, Lys170, Lys147, Asp137, Val139
**PRM1**				
536	Oolonghomobisflavan A	-26.58	2.42	Leu46, Leu54, Ile56, Ile84, Val92, Met107, Glu108, Lys91, Pro38, Leu39, Leu87, Asn90, Phe105, Ile71, Gln120, Leu58, Val41, Lys60, Lys69, Tyr42, Leu122

## DISCUSSION

The advances in a recent day molecular biology provides ample information suggesting that phenotypes are actually a result of several genes that works in a network. Reproduction is a complex biological process that involves a number of genes and pathways to work together in a coherent way to bring out the desired phenotypes or germ cells capable of fertilization. WGCNA has been widely used to find out candidate or signature genes for disease condition [21]. We have used this state of the art analysis tool to find out reproductive driver genes across *Bos taurus* and *Sus scrofa*. Such an analysis has a wide application in animal reproductive biology where one can target specific set of genes as per own desire to generate desired phenotypes for higher reproductive performance, fertility or sex ratio control.

The gene enrichment in co-expression network of genes in present study demonstrates 66 different biological processes including genes related to immunity indicating its connected role in reproduction. It is worth noting that the immunity genes co-express with reproductive genes provide a safe microenvironment for the sperm to develop as well as to fertilize oocyte in germ free environment. Another probable role of those genes could be assumed as generating a suitable environment in female reproductive tract to select specific X or Y bearing spermatozoa [22]. Such genes can be exploited in favor of female or male sex ratio control.

There are mechanisms that favor the X chromosome bearing spermatozoa to fertilize oocyte. One such mechanism is that at time of spermiogenesis, the tail formation of sperm where certain gene expression benefits X bearing spermatozoa can make the female ratio higher than expected 1:1 [5, 23]. In present study PAFAH1B1, PPP2R2B and PRM1 is involved with spermatid development, where PAFAH1B1 have a role with spermiogenesis and sperm maturation. At this stage PAFAH1B1 is responsible for tail formation where it can differentially benefit X bearing spermatozoa to develop tail that can increase the ratio of female from normal 1:1.

It is proposed in Mendel's first law that an XY cross will produce a 1:1 male to female ratio in sexually reproducing organisms as evolutionary forces favor parents that spend equal amount of resources on both sexes and an imbalance in this ratio will immediately counter balance the effect in favor of under-presented sex [24]. It has been observed in a population of metazoans that it transmits its sex chromosomes unequally that cause broods to produce highly skewed sex ratio and was termed as a result of asymmetric spermatocyte division [23]. There are three possible ways for distorted sex ratio in genetic terms 1) non random sex chromosome segregation at meiotic division [25] 2) post meiotic differences in haploid gametes causes sperm specific function differences in response to sex chromosomes [26] or 3) sex specific embryonic lethality caused by post zygotic mechanisms [27]. Recently two spermatogenesis based modifications to the cellular program are reported in nematode Rhabditis sp. sB347 that sire >5% male progeny. Firstly the meiosis is modified where sister chromatids of unpaired X chromosome separate prematurely at meiosis 1 stage, and secondly during anaphase II of meiosis II, cellular components that are essential for sperm motility are partitioned exclusively to X-bearing sperm [23]. In present study, we found that the PRM1, PPP2R2B and PAFAH1B1 are associated with spermatogenesis specially the LiSH domain containing protein PAFAH1B1 have role in chromosome segregation and can be used as target for sex ratio increase in favor of female. More interestingly several genes that are associated with motility are co-expressed with reproductive driver genes suggesting its combined role in producing healthy sperm capable of fertilization; and those genes can be made exclusively partitioned proteins responsible for motility to X bearing spermatozoa in controlled breeding programs. Furthermore, specific alterations in gene expression can partition the components of essential sperm motility exclusively to X or Y chromosome hence change normal sex ratio.

Sperm needs to undergo many post meiotic changes of the spermatogenic process before it starts its journey into the reproductive tract of female. The haploid spermatid undergo striking morphological changes including restructuring and compaction of its chromatin material. This makes the almost complete substitution of histone based nucleosomal and histone based structure with protamine based structure. Protamine protects the paternal genetic material and remains key factor in male fertility [28]. In our present study PRM1 is differentially expressed and is present in reproductive module and hence is an important reproductive driver gene. The consequence of mutations in protamine based chromatin can result in infertility in mammals, in our results, the dbSNP search shows no SNP records in PRM1, although we found two structural variations from Database of Genomic Variants (DGV) showing mutations in PRM1 gene is highly protected to safeguard paternal genetic material.

To further elucidate the important roles of PRM1, PPP2R2B, PAFAH1B1 we made genetic analysis of these genes. We made a phylogenetic tree, predicted important domains, carried out epigenetic analysis and mutation analysis. Furthermore, to regulate the expression of these gene we performed protein docking so that we can recommend chemicals or food items that are beneficial or harmful for reproduction.

## MATERIALS AND METHODS

The Holstein sperm was separated by flow cytometry and after achieving purity of 95% and stored at -80 C. Then we extracted RNA according to manual performed RNA quality testing and then make the microarray geneChip.

### Data and method

### Data collection and pre-processing

The hybridized microarrays results in images that were used to generate datasets which were pre processed to get meaningful data sets for further analysis. A particular type of preprocessing is termed as normalization that accounts for systematic difference across different datasets. To identify reproductive driver genes, two datasets were included in present study: 1) X and Y chromosome Affimetrix transcriptome data of *Bos taurus* generated in our lab (only one sample for each chromosome). 2) NCBI raw transcriptome data of *Sus scrofa* with accession number of GSE47139 [22] was downloaded from GEO website for analysis (a total of eight samples in GSE47139, 4 X and 4 Y chromosome Affimetrix transcriptomes of *Sus scrofa*.). Both datasets were pre-processed by using R-language Affy package [29], including background correction and normalized simultaneously. The data sets are all normalized by using Limma package in R language.

### Consensus modules identification of *Bos taurus* and *Sus scrofa*

We constructed co-expression network and mined reproductive related modules in *Bos taurus* and *Sus scrofa* respectively, based on weighed gene co-expression network analysis (WGCNA) algorithm, which is a typical system biology algorithm for constructing gene co-expression network [30]. We extracted overlapped genes in *Bos taurus* and *Sus scrofa* for WGCNA, the network building and module identification process steps in *Bos taurus* transcriptome were as follows:

#### Definition of gene expression correlation matrix

Correlation coefficient of gene m and gene n was defined as:

S_mn_=|cor_(m,n)_|, We calculated expression correlation coefficient of every two genes to make a whole correlation coefficient matrix.

#### Definition of adjacency function

WGCNA used power index adjacency function as measurement of correlated indicators, the function was defined as:

a_mn_, where, a_mn_=power(S_mn_,β), then determined weighted factor β, we choose β above 0.9 as a power cutoff.

#### Node dissimilarity measurement

The adjacency matrix amn was transformed into a topological matrix Ω=wmn, where

$${\text{w}}_{\text{mn}}=\frac{l_{\text{mn}}+{\text{a}}_{\text{mn}}}{\min\{{\text{k}}_{\text{m}},{\text{k}}_{\text{n}}\}+1-{\text{a}}_{\text{mn}}}$$

l_mn_ referred to adjacency coefficients sum of all connected nodes, k_m_ represented connection sum of gene m, dissimilarity of genes was defined as d_mn_, d_mn_ = 1 - w_mn_.

#### Module identification

We constructed hierarchical clustering tree based on d_mn_ defined before, tree branches represented different gene modules.

#### Screening consensus modules in *Sus scrofa*

We also identified reproductive related modules in *Sus scrofa* based on WGCNA with same processes and cutoffs as in *Bos taurus* described above. Then we calculated overlapped genes of each consensus modules in *Bos taurus* and *Sus scrofa*, the selected overlapped genes in consensus modules were used for further analysis.

### Gene significance analysis

Filtered expression data of *Bos taurus* were analyzed with the test method in Limma package [31], implemented in R language, Bioconductor project. Meanwhile, fold change between groups were also calculated, because each sample may show intrinsic individual variability, the threshold for determining the fold change (FC) was set at 1.5. Significantly differentially expressed genes (DEG) were defined with the cutoff of p value <0.05 and |log2FC|>0.585. Then we selected shared genes by comparing DEGs and selected overlapped genes in consensus modules. The shared genes were used for further analyses.

### Construction of co-expression network of shared genes

We calculated gene expression correlation coefficient of every two selected shared genes, and kept gene pairs with correlation coefficient higher than 0.8 to construct gene co-expression network. Network was visualized by Cytoscape 2.8.0 software [32].

### Functional and KEGG pathway analysis of the network genes

To further analyze the functions of network genes, we enriched genes in the co-expression network to Gene Ontology (GO) and Kyoto Encyclopedia of Genes and Genomes (KEGG) pathways by using clusterProfiler [33] with a cutoff of p lower than 0.05.

### Related miRNA and TF search

We searched significantly related TF by using The Database for Annotation, Visualization and Integrated Discovery (DAVID) v6.8 [34], and extracted related miRNAs of genes in co-expression network from miRTarBase (2016 version) [35]. Then constructed TF/miRNA -targeted co-expression network by integrating miRNA/TF and co-expression information.

### Genetic analysis of vital reproduction genes across *Bos taurus* and *Sus scrofa*

Phylogenetic tree was made using Mega 6 after aligning the downloaded sequences in ClustalW.We searchedwith InterProScan [19] in EBI, we identify CpG islands using Methprimer [20], and finally searched SNPs and CNVs from dbSNP and DGV respectively.

### Protein docking analysis of vital reproduction genes

Molecular docking analysis were performed using MOE (Molecular Operating Environment) software [36]. Protein structures of vitally important genes were downloaded from PDB (Protein Data Bank) [37] and optimized by minimizing their energies with parameters (Force Field: AMBER99, Gradient: 0.05). The minimized structures were used as the receptor protein for docking. MOE site finder tool was used to find out the active site where ligands can bind. To select a suitable ligand, selected proteins were docked against ready-to-dock library of MPD3 database [38] contacting 2500 potential compounds in 3D ready to dock format. MOE docking program with parameters (Rescoring function: London dG, Placement: Triangle matcher, Retain: 10, Refinement: Force field, Rescoring 2: London dG) was used to bind the selected ligands with proteins.

## SUPPLEMENTARY MATERIALS FIGURES AND TABLES

















## Abbreviations

DEGs: Differentially expressed genes
KEGG: Kyoto Encyclopedia of Genes and Genomes
BP: biological process
GO: Gene Ontology
WGCNA: Weighted Gene Co-Expression Network Analysis
PDB: Protein Data Bank
MOE: Molecular Operating Environment
TF: Trancription Factor
