# Supplementary material for: Analysis of Bos taurus and Sus scrofa X and Y chromosome transcriptome highlights reproductive driver genes
Source: Oncotarget. 2017 Apr 13;8(33):54416–33. doi: 10.18632/oncotarget.17081 (PMC5589591; doi:10.18632/oncotarget.17081)
Supplement: Supplementary file 1 [file oncotarget-08-54416-s001.pdf]

Analysis of *Bos taurus* and *Sus scrofa* X and Y chromosome transcriptome highlights reproductive driver genes

SUPPLEMENTARY FIGURE AND TABLES

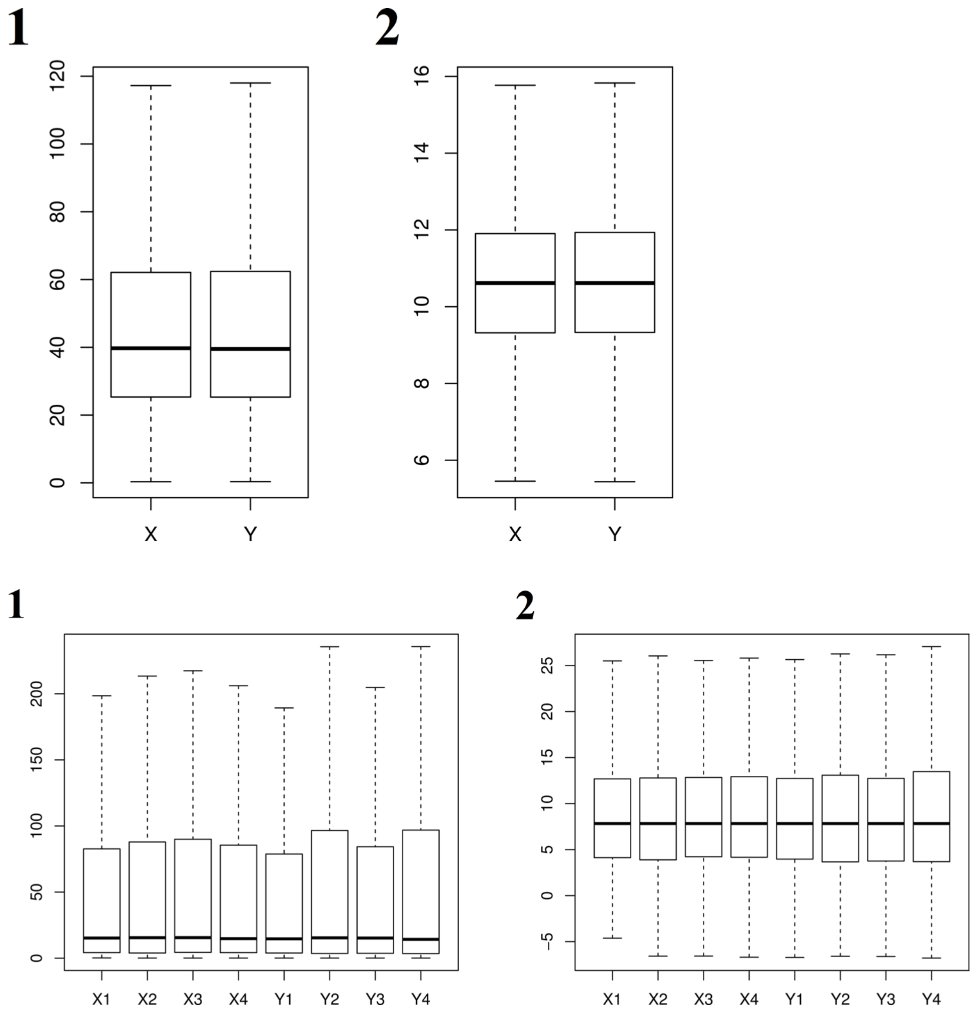

Supplementary Figure 1: Expression data before and after normalization of *Bos taurus* X and Y spematozoa (Supplementary Figure 1A) Sus scrofa GSE47139 (Supplementary Figure 1B).

Supplementary Table 1: Expression data before and after normalization

See Supplementary File 1

Supplementary Table 2: Shared homologous gene list of *Bos taurus* and *Sus scrofa* in expression data

See Supplementary File 2

Supplementary Table 3: Corresponding genes and their module color in *Bos taurus* and *Sus scrofa*

See Supplementary File 3

Supplementary Table 4: Gene list of significantly overlapped genes in consensus modules

See Supplementary File 4

Supplementary Table 5: Differentially expressed genes (DEGs) in *Bos taurus*

See Supplementary File 5

Supplementary Table 6: The dual featured expressed gene list in X and Y transcriptome

See Supplementary File 6

Supplementary Table 7: Correlation coefficient matrix of selected genes

See Supplementary File 7

Supplementary Table 8: Complete list of GO annotations

See Supplementary File 8

Supplementary Table 9: miRNA list targeted genes in co-expression network

SCD	MIRT053989	bta-miR-181a	Bos taurus
LDLR	MIRT053868	bta-miR-17-5p	Bos taurus
LDLR	MIRT053942	bta-miR-146b	Bos taurus
LDLR	MIRT053926	bta-miR-146a	Bos taurus
PLAU	MIRT053969	bta-miR-181a	Bos taurus

Supplementary Table 10: Sequences of PPP2R2B, PRM1 and PAFAH1B1 in *Bos taurus* and *Sus scrofa*

>PPP2R2B-*Bos taurus*
MEEDIDTRKINNSFLRDHSYATEADIISTVEFNHTGELLATGDKGGRVVIFQREQESKNQVHRRGEYNVYSTFQSH
EPEFDYLKSLEIEEKINKIRWLPQQNAAYFLLSTNDKTVKLWKVSERDKRPEGYNLKDEEGRLRDPATITTLRVPV
LRPMDLMVEATPRRVFANAHTYHINSISVNSDYETYMSADDLRINLWNFEITNQSFNIVDIKPANMEELTEVITAA
EFHPHHCNTFVYSSSKGTIRLCDMRASALCDRHTKFFEEPEDPSNRSFFSEIISSISDVKFSHSGRYIMTRDYLTVKV
WDLNMENRPIETYQVHDYLRSKLCSLYENDCIFDKFECVWNGSDSVIMTGSYNNFFRMFDRNTKRDVTLEASRE
NSKPRAILKPRKVCVGGKRRKDEISVDSLDFSKKILHTAWHPSENIIAVAATNNLYIFQDKVN

>PRM1-*Bos taurus*
MARYRCCLTHSGSRCRRRRRRRCRRRRRRFGRRRRRRVCCRRYTVIRCTRQ

>PAFAH1B1-*Bos taurus*
MVLSQRQRDELNRAIADYLRSNGYEEAYSVFKKEAELDMNEELDKKYAGLLEKKWTSVIRLQKKVMELESKLN
EAKEEFTSGGPLGQKRDPKEWIPRPPEKYALSGHRSPVTRVIFHPVFSVMVSASEDATIKVWDYETGDFERTLKG
HTDSVQDISFDHSGKLLASCSADMTIKLWDFQGFECIRTMHGHDHNVSSVAIMPNGDHIVSASRDKTIKMWEVQ
TGYCVKTFTGHREWVRMVRPNQDGTLIASCSNDQTVRVWVVATKECKAELREHEHVVECISWAPESSYSSISEAT
GSETKKSGKPGPFLLSGSRDKTIKMWDVSTGMCLMTLVGHDNWVRGVLFHSGGKFILSCADDKTLRVWDYKN
KRCMKTLNAHEHFVTSLDFHKTAPYVVTGSVDQTVKVWECR

>PAFAH1B1-*Sus scrofa*
MVLSQRQRDELNRAIADYLRSNGYEEAYSVFKKEAELDMNEELDKKYAGLLEKKWTSVIRLQKKVMELESKLN
EAKEEFTSGGPLGQKRDPKEWIPRPPEKYALSGHRSPVTRVIFHPVFSVMVSASEDATIKVWDYETGDFERTLKG
HTDSVQDISFDHSGKLLASCSADMTIKLWDFQGFECIRTMHGHDHNVSSVAIMPNGDHIVSASRDKTIKMWEVQ
TGYCVKTFTGHREWVRMVRPNQDGTLIASCSNDQTVRVWVVATKECKAELREHEHVVECISWAPESSYSSISEAT
GSETKKSGKPGPFLLSGSRDKTIKMWDVSTGMCLMTLVGHDNWVRGVLFHSGGKFILSCADDKTLRVWDYKN
KRCMKTLNAHEHFVTSLDFHKTAPYVVTGSVDQTVKVWECR

>PRM1-*Sus scrofa*
MARYRCCRSHSRSRCRPRRRRCRRRRRRCCPRRRRAVCCRRYTVIRCRRC

>PPP2R2B-*Sus scrofa*
MEEDIDTRKINNSFLRDHSYATEADIISAVEFNHTGELLATGDKGGRVVIFQREQESKNQVHRRGEYNVYSTFQSH
EPEFDYLKSLEIEEKINKIRWLPQQNAAYFLLSTNDKTVKLWKVSERDKRPEGYNLKDEEGRLRDPATITTLRVPV
LRPMDLMVEATPRRVFANAHTYHINSISVNSDYETYMSADDLRINLWNFEITNQSFNIVDIKPANMEELTEVITAA
EFHPHHCNTFVYSSSKGTIRLCDMRASCVTGTPNFFFEEPEDPSNRSFFSEIISSISDVKFSHSGRYIMTRDYLTVKV
WDLNMENRPIETYQVSVFLRGKLISLHIKNKCMIAKDDARVDGSDSVIMTGSYNNFFRMFDRNTKRDVTLEASR
ENSKPRAILKPRKVCVGGKRRKDEISVDSLDFSKKILHTAWHPSENIIAVAATNNLYIFQDKVN
